# *N*,*N*-Dimethyl-4-[(2-pyrid­yl)diazen­yl]aniline

**DOI:** 10.1107/S1600536810025754

**Published:** 2010-07-07

**Authors:** Nararak Leesakul, Suthirat Yoopensuk, Chaveng Pakawatchai, Saowanit Saithong, Kanidtha Hansongnern

**Affiliations:** a Department of Chemistry, Faculty of Science, Prince of Songkla University, Hat Yai, Songkhla 90112, Thailand

## Abstract

The title compound, C_13_H_14_N_4_, adopts a *trans* configuration about the azo bond. There is a dihedral angle of 12.18 (7)° between the pyridine and benzene rings and the mean plane of the dimethyl­amino substituent is twisted by 6.1 (2)° relative to the benzene ring. In the crystal, weak inter­molecular C—H⋯N hydrogen bonds result in a zigzag arrangement along [010].

## Related literature

For applications of azo compounds in textile coloring and photovoltaic frameworks, see: Millington *et al.* (2007 ▶). For the synthesis of similar compounds, see: Krause & Krause (1980 ▶). For the X-ray structures of protonated 2-(phenylazo)pyridine (azpy), a similar compound, and chelating complexes, see: Panneerselvam *et al.* (2000 ▶); Peacock *et al.* (2007 ▶); Ohashi *et al.* (2003 ▶). For the X-ray structures of complexes with the title compound, see: Dougan *et al.* (2006 ▶); Li *et al.* (2001 ▶);
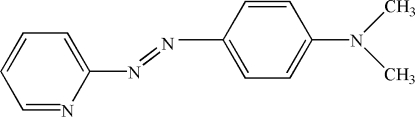


## Experimental

### 

#### Crystal data

C_13_H_14_N_4_
*M* *_r_* = 226.28
Monoclinic, 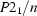

*a* = 6.2322 (4) Å
*b* = 19.9353 (11) Å
*c* = 9.6404 (6) Å
β = 96.003 (1)°
*V* = 1191.16 (13) Å^3^
*Z* = 4
Mo *K*α radiation
μ = 0.08 mm^−1^
*T* = 293 K
0.28 × 0.26 × 0.06 mm

#### Data collection

Bruker SMART APEX CCD area-detector diffractometer
Absorption correction: multi-scan (*SADABS*; Bruker, 2003 ▶) *T*_min_ = 0.918, *T*_max_ = 1.000
12811 measured reflections
2101 independent reflections
1754 reflections with *I* > 2σ(*I*)
*R*_int_ = 0.023

#### Refinement

*R*[*F* ^2^ > 2σ(*F* ^2^)] = 0.038
*wR*(*F* ^2^) = 0.108
*S* = 1.04
2101 reflections
156 parameters
H-atom parameters constrained
Δρ_max_ = 0.12 e Å^−3^
Δρ_min_ = −0.19 e Å^−3^

### 

Data collection: *SMART* (Bruker, 1998 ▶); cell refinement: *SAINT* (Bruker, 2003 ▶); data reduction: *SAINT*; program(s) used to solve structure: *SHELXTL* (Sheldrick, 2008 ▶); program(s) used to refine structure: *SHELXTL*; molecular graphics: *Mercury* (Macrae *et al.*, 2008 ▶); software used to prepare material for publication: *Mercury* and *SHELXTL*.

## Supplementary Material

Crystal structure: contains datablocks I, global. DOI: 10.1107/S1600536810025754/fj2313sup1.cif

Structure factors: contains datablocks I. DOI: 10.1107/S1600536810025754/fj2313Isup2.hkl

Additional supplementary materials:  crystallographic information; 3D view; checkCIF report

## Figures and Tables

**Table 1:** Hydrogen-bond geometry (Å, °)

*D*—H⋯*A*	*D*—H	H⋯*A*	*D*⋯*A*	*D*—H⋯*A*
C1—H1⋯N3^i^	0.93	2.58	3.4516 (18)	157

Symmetry code: (i) 
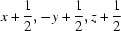
.

**Fig. 1.**
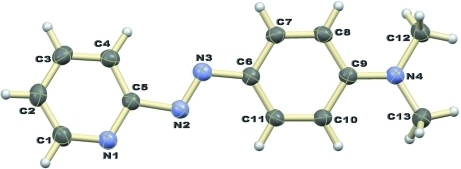
The molecular structure of 4-(2-pyridylazo)-N,N-dimethylaniline. Thermal ellipsoids are shown at 50% probability level.

**Fig. 2.**
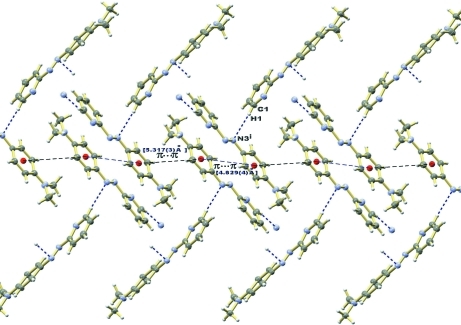
The packing interactions of 4-(2-pyridylazo)-N,N-dimethylaniline. Symmetry code: (i) x + 1/2, -y + 1/2, Z+1/2.

## supplementary crystallographic information

### Comment

Compounds existing of azo-imine (–N—C—N=N–) moieties have been extensively used as the powerful ligands of transition metal complexes owing to their great σ-donor and π-acceptor properties. Applications of azo compounds are on textile coloring and photovoltaic frameworks (Millington *et al.*, 2007). As being a part of our studies on dye-sensitized solar cell, here we report the X-ray structure of the title compound synthesized by modified 2-phenylazopyridine (azpy) synthetic pathway (Krause *et al.*, 1980).

The molecule is almost coplanar as shown in Fig. 1. Torsion angle of pyridine-azo-phenyl atoms,*C*(5)—N(2)—N(3)—C(6), is -179.68 (10)*°*. The mean planes of pyridine and phenyl rings deviate for 12.18 (7)*°*. The N(py) atom exists in *trans*-form with respect to the N(azo) atom attached to the phenyl ring. It is different from that observed in protonated azpy (Panneerselvam *et al.*, 2000) and chelating complexes (Peacock *et al.*, 2007; Ohashi *et al.*, 2003) in which the *cis*- configurations are observed. The N=N bond distance of free dmazpy [1.2566 (16) Å] is long in comparison with its chelating Os(II) complex [1.301 (4) Å] (Peacock *et al.*, 2007) because of π-backbonding interaction. The methylaniline substituent plane is slightly deviated from phenyl ring with dihedral angle of 6.1 (2)*°*. In the crystal structure, the weak hydrogen bonds are found at the N(azo) atom attached to the phenyl ring, C(1)—H(1)···N(3) [C···N = 3.4516 (18) Å]. Intermolecular π-π stacking interactions occur between adjacent phenyl rings. The centroid-centroid distances are found the alternated distances of 5.317 (3) Å and 4.629 (4) Å sequences parallel to the *c* axis. All these interactions link the molecules into a zigzag orientation parallel to [010]. The packing interactions are shown in Fig.2.

### Experimental

2-Aminopyridine (0.50 g, 5 mmol) was dissolved in 5 ml benzene. The solution was heated at 80 *°*C for 10 minutes. Then 6 ml of 25 *M* NaOH was slowly added into the 2-aminopyridine solution. *N,N*-dimethyl-1,4-nitrosoaniline (0.75 g, 5 mmol) was gradually added to the warm solution mixture. An additional 5 ml of benzene was put into the solution mixture which was then refluxed for 9 h. The reaction mixture was filtered and extracted with benzene. Red powder was purified by silica gel column chromatography using mixture of hexane and ethylacetate as an eluent. Recrystallization at room temperature in 3:2 hexane: methanol mixture yielded red crystals. The dmazpy melting point is 104–105 *°*C. Anal. Calcd for dmazpy: C, 69.00; H, 6.23; N, 24.76. Found: C, 69.85; H, 6.34; N, 23.81. ES—MS: m/*z* 227(MH^+^, 100%).

### Refinement

The structures were solved by direct methods refined by a full-matrix least-squares procedure based on *F*^2^. All Hydrogen atoms were placed in geometrically idealized positions and refined isotropically with a riding model for both of C-*sp*^2^ and [C—H = 0.93 Å and with *U*_iso_(H) = 1.2*U*_eq_(C)] and C-*sp*^3 ^[C—H = 0.96 Å and with *U*_iso_(H) = 1.5*U*_eq_(C)].

### Crystal data

**Table:**

C_13_H_14_N_4_	*F*(000) = 480
*M**_r_* = 226.28	*D*_x_ = 1.262 Mg m^−^^3^
Monoclinic, *P*2_1_/*n*	Mo *K*α radiation, λ = 0.71073 Å
Hall symbol: -P 2yn	Cell parameters from 3335 reflections
*a* = 6.2322 (4) Å	θ = 2.4–25.4°
*b* = 19.9353 (11) Å	µ = 0.08 mm^−^^1^
*c* = 9.6404 (6) Å	*T* = 293 K
β = 96.003 (1)°	Block, colorless
*V* = 1191.16 (13) Å^3^	0.28 × 0.26 × 0.06 mm
*Z* = 4	

### Data collection

**Table:**

Bruker APEX CCD area-detector diffractometer	2101 independent reflections
Radiation source: fine-focus sealed tube	1754 reflections with *I* > 2σ(*I*)
graphite	*R*_int_ = 0.023
φ and ω scans	θ_max_ = 25.0°, θ_min_ = 2.0°
Absorption correction: multi-scan (*SADABS*; Bruker, 2003)	*h* = −7→7
*T*_min_ = 0.918, *T*_max_ = 1.000	*k* = −23→23
12811 measured reflections	*l* = −11→11

### Refinement

**Table:**

Refinement on *F*^2^	Primary atom site location: structure-invariant direct methods
Least-squares matrix: full	Secondary atom site location: difference Fourier map
*R*[*F*^2^ > 2σ(*F*^2^)] = 0.038	Hydrogen site location: inferred from neighbouring sites
*wR*(*F*^2^) = 0.108	H-atom parameters constrained
*S* = 1.04	*w* = 1/[σ^2^(*F*_o_^2^) + (0.057*P*)^2^ + 0.1546*P*] where *P* = (*F*_o_^2^ + 2*F*_c_^2^)/3
2101 reflections	(Δ/σ)_max_ < 0.001
156 parameters	Δρ_max_ = 0.12 e Å^−^^3^
0 restraints	Δρ_min_ = −0.18 e Å^−^^3^

### Special details

**Table:**

Geometry. All e.s.d.'s (except the e.s.d. in the dihedral angle between two *l.s. *planes) are estimated using the full covariance matrix. The cell e.s.d.'s are taken into account individually in the estimation of e.s.d.'s in distances, angles and torsion angles; correlations between e.s.d.'s in cell parameters are only used when they are defined by crystal symmetry. An approximate (isotropic) treatment of cell e.s.d.'s is used for estimating e.s.d.'s involving *l.s.* planes.
Refinement. Refinement of *F*^2^ against all reflections. The weighted *R*-factor *wR* and goodness of fit *S* are based on *F*^2^, conventional *R*-factors *R* are based on *F*, with *F* set to zero for negative *F*^2^. The threshold expression of *F*^2^ > σ(*F*^2^) is used only for calculating *R*-factors(gt) *etc*. and is not relevant to the choice of reflections for refinement. *R*-factors based on *F*^2^ are statistically about twice as large as those based on *F*, and *R*- factors based on all data will be even larger.

### Fractional atomic coordinates and isotropic or equivalent isotropic displacement parameters (Å^2^)

**Table:**

	*x*	*y*	*z*	*U*_iso_*/*U*_eq_	
N1	0.26625 (18)	0.22136 (6)	0.68133 (12)	0.0540 (3)	
C1	0.2173 (3)	0.27093 (7)	0.76510 (16)	0.0609 (4)	
H1	0.3306	0.2956	0.8102	0.073*	
C2	0.0116 (3)	0.28823 (7)	0.78947 (16)	0.0622 (4)	
H2	−0.0136	0.3234	0.8490	0.075*	
C3	−0.1564 (2)	0.25173 (8)	0.72277 (15)	0.0619 (4)	
H3	−0.2983	0.2620	0.7363	0.074*	
C4	−0.1119 (2)	0.20013 (7)	0.63609 (15)	0.0547 (4)	
H4	−0.2227	0.1746	0.5906	0.066*	
C5	0.1016 (2)	0.18679 (6)	0.61771 (13)	0.0449 (3)	
N2	0.16987 (18)	0.13418 (5)	0.53090 (11)	0.0498 (3)	
N3	0.01419 (17)	0.10823 (5)	0.45640 (11)	0.0487 (3)	
C6	0.0660 (2)	0.05591 (6)	0.36836 (13)	0.0450 (3)	
C7	−0.1052 (2)	0.02897 (7)	0.28271 (15)	0.0516 (3)	
H7	−0.2431	0.0459	0.2879	0.062*	
C8	−0.0770 (2)	−0.02177 (7)	0.19098 (14)	0.0508 (3)	
H8	−0.1955	−0.0386	0.1352	0.061*	
C9	0.1288 (2)	−0.04879 (6)	0.17993 (13)	0.0456 (3)	
C10	0.3025 (2)	−0.02146 (7)	0.26841 (15)	0.0530 (4)	
H10	0.4408	−0.0383	0.2644	0.064*	
C11	0.2711 (2)	0.02924 (7)	0.35976 (14)	0.0511 (3)	
H11	0.3881	0.0462	0.4169	0.061*	
N4	0.15861 (18)	−0.09932 (6)	0.08935 (12)	0.0542 (3)	
C12	−0.0229 (2)	−0.13009 (7)	0.00681 (16)	0.0597 (4)	
H12A	−0.1279	−0.1441	0.0670	0.090*	
H12B	0.0260	−0.1684	−0.0415	0.090*	
H12C	−0.0871	−0.0982	−0.0597	0.090*	
C13	0.3730 (2)	−0.12209 (9)	0.06728 (19)	0.0733 (5)	
H13A	0.4497	−0.0864	0.0272	0.110*	
H13B	0.3624	−0.1598	0.0051	0.110*	
H13C	0.4490	−0.1351	0.1549	0.110*	

### Atomic displacement parameters (Å^2^)

**Table:**

	*U*^11^	*U*^22^	*U*^33^	*U*^12^	*U*^13^	*U*^23^
N1	0.0499 (7)	0.0566 (7)	0.0556 (7)	−0.0048 (5)	0.0061 (5)	−0.0065 (6)
C1	0.0631 (9)	0.0583 (9)	0.0613 (9)	−0.0094 (7)	0.0064 (7)	−0.0099 (7)
C2	0.0747 (10)	0.0535 (8)	0.0597 (9)	0.0077 (8)	0.0127 (8)	−0.0043 (7)
C3	0.0532 (8)	0.0707 (10)	0.0621 (9)	0.0137 (7)	0.0086 (7)	0.0022 (8)
C4	0.0480 (8)	0.0636 (9)	0.0516 (8)	−0.0012 (6)	0.0009 (6)	0.0009 (7)
C5	0.0477 (7)	0.0448 (7)	0.0419 (7)	0.0004 (6)	0.0029 (6)	0.0048 (5)
N2	0.0488 (6)	0.0510 (6)	0.0492 (6)	−0.0023 (5)	0.0038 (5)	−0.0007 (5)
N3	0.0507 (7)	0.0462 (6)	0.0484 (6)	−0.0008 (5)	0.0017 (5)	0.0041 (5)
C6	0.0483 (7)	0.0421 (7)	0.0447 (7)	−0.0007 (5)	0.0049 (6)	0.0049 (5)
C7	0.0416 (7)	0.0521 (8)	0.0612 (8)	0.0003 (6)	0.0052 (6)	0.0004 (7)
C8	0.0420 (7)	0.0517 (8)	0.0574 (8)	−0.0063 (6)	−0.0003 (6)	−0.0019 (6)
C9	0.0453 (7)	0.0444 (7)	0.0473 (7)	−0.0048 (6)	0.0054 (6)	0.0034 (5)
C10	0.0408 (7)	0.0558 (8)	0.0619 (8)	0.0011 (6)	0.0028 (6)	−0.0051 (7)
C11	0.0464 (8)	0.0528 (8)	0.0524 (8)	−0.0038 (6)	−0.0032 (6)	−0.0016 (6)
N4	0.0472 (7)	0.0551 (7)	0.0601 (7)	−0.0034 (5)	0.0048 (5)	−0.0105 (6)
C12	0.0590 (9)	0.0583 (9)	0.0612 (9)	−0.0100 (7)	0.0036 (7)	−0.0086 (7)
C13	0.0571 (9)	0.0797 (11)	0.0834 (11)	0.0040 (8)	0.0089 (8)	−0.0265 (9)

### Geometric parameters (Å, °)

**Table:**

N1—C5	1.3311 (16)	C7—H7	0.9300
N1—C1	1.3316 (18)	C8—C9	1.4052 (18)
C1—C2	1.371 (2)	C8—H8	0.9300
C1—H1	0.9300	C9—N4	1.3586 (17)
C2—C3	1.378 (2)	C9—C10	1.4156 (18)
C2—H2	0.9300	C10—C11	1.3678 (19)
C3—C4	1.372 (2)	C10—H10	0.9300
C3—H3	0.9300	C11—H11	0.9300
C4—C5	1.3863 (19)	N4—C13	1.4477 (18)
C4—H4	0.9300	N4—C12	1.4494 (17)
C5—N2	1.4337 (16)	C12—H12A	0.9600
N2—N3	1.2566 (15)	C12—H12B	0.9600
N3—C6	1.4035 (16)	C12—H12C	0.9600
C6—C7	1.3870 (18)	C13—H13A	0.9600
C6—C11	1.3951 (19)	C13—H13B	0.9600
C7—C8	1.3668 (19)	C13—H13C	0.9600
			
C5—N1—C1	116.70 (12)	C9—C8—H8	119.6
N1—C1—C2	124.61 (14)	N4—C9—C8	121.36 (12)
N1—C1—H1	117.7	N4—C9—C10	121.68 (12)
C2—C1—H1	117.7	C8—C9—C10	116.95 (12)
C1—C2—C3	117.76 (14)	C11—C10—C9	121.37 (12)
C1—C2—H2	121.1	C11—C10—H10	119.3
C3—C2—H2	121.1	C9—C10—H10	119.3
C4—C3—C2	119.19 (14)	C10—C11—C6	120.90 (12)
C4—C3—H3	120.4	C10—C11—H11	119.6
C2—C3—H3	120.4	C6—C11—H11	119.6
C3—C4—C5	118.67 (13)	C9—N4—C13	121.16 (11)
C3—C4—H4	120.7	C9—N4—C12	121.01 (11)
C5—C4—H4	120.7	C13—N4—C12	117.77 (12)
N1—C5—C4	123.06 (12)	N4—C12—H12A	109.5
N1—C5—N2	112.72 (11)	N4—C12—H12B	109.5
C4—C5—N2	124.21 (12)	H12A—C12—H12B	109.5
N3—N2—C5	112.11 (11)	N4—C12—H12C	109.5
N2—N3—C6	116.04 (11)	H12A—C12—H12C	109.5
C7—C6—C11	118.00 (12)	H12B—C12—H12C	109.5
C7—C6—N3	115.89 (12)	N4—C13—H13A	109.5
C11—C6—N3	126.11 (12)	N4—C13—H13B	109.5
C8—C7—C6	121.93 (13)	H13A—C13—H13B	109.5
C8—C7—H7	119.0	N4—C13—H13C	109.5
C6—C7—H7	119.0	H13A—C13—H13C	109.5
C7—C8—C9	120.85 (12)	H13B—C13—H13C	109.5
C7—C8—H8	119.6		
			
C5—N1—C1—C2	0.0 (2)	N3—C6—C7—C8	179.35 (12)
N1—C1—C2—C3	0.1 (2)	C6—C7—C8—C9	0.0 (2)
C1—C2—C3—C4	0.2 (2)	C7—C8—C9—N4	179.85 (12)
C2—C3—C4—C5	−0.5 (2)	C7—C8—C9—C10	0.5 (2)
C1—N1—C5—C4	−0.41 (19)	N4—C9—C10—C11	−179.74 (12)
C1—N1—C5—N2	−179.47 (11)	C8—C9—C10—C11	−0.4 (2)
C3—C4—C5—N1	0.7 (2)	C9—C10—C11—C6	−0.2 (2)
C3—C4—C5—N2	179.60 (12)	C7—C6—C11—C10	0.7 (2)
N1—C5—N2—N3	−170.52 (11)	N3—C6—C11—C10	−179.24 (12)
C4—C5—N2—N3	10.44 (17)	C8—C9—N4—C13	173.23 (14)
C5—N2—N3—C6	−179.68 (10)	C10—C9—N4—C13	−7.5 (2)
N2—N3—C6—C7	−177.82 (11)	C8—C9—N4—C12	−3.9 (2)
N2—N3—C6—C11	2.14 (18)	C10—C9—N4—C12	175.44 (12)
C11—C6—C7—C8	−0.6 (2)		

### Hydrogen-bond geometry (Å, °)

**Table:**

*D*—H···*A*	*D*—H	H···*A*	*D*···*A*	*D*—H···*A*
C1—H1···N3^i^	0.93	2.58	3.4516 (18)	157

Symmetry codes: (i) *x*+1/2, −*y*+1/2, *z*+1/2.
